# Benefits of horseback riding for neurotypical children and adolescents: a scoping review

**DOI:** 10.1590/2317-1782/e20240083en

**Published:** 2025-03-31

**Authors:** Flaviana Gomes da Silva, Danielle Diniz de Paula, Luciana Mendonça Alves, Juliana Nunes Santos

**Affiliations:** 1 Departamento da Pós-graduação em Ciências Fonoaudiológicas, Faculdade de Medicina, Universidade Federal de Minas Gerais – UFMG - Belo Horizonte (MG), Brasil.; 2 Instituto de Ciência e Tecnologia – ICTIN, Universidade Federal de Lavras – UFLA - Lavras (MG), Brasil.

**Keywords:** Equine-Assisted Therapy, Child or Adolescent Development, Cognition, Socialization, Child Behavior, Language

## Abstract

**Purpose:**

To investigate evidence of horse riding in the development of language, cognition, social, emotional, and behavioral aspects in neurotypical children and adolescents.

**Research strategies:**

Search in the databases of LILACS, MEDLINE, Web of Science, EMBASE, Scopus, and grey literature, without date or language restrictions. Registration in the Open Science Framework (OSF), under number DOI 10.17605/OSF.IO/32ETZ. For search strategies: “Equine-Assisted Therapy”, “Child or Adolescent Development”, Cognition, Socialization, and “Child Behavior”.

**Selection criteria:**

Randomized clinical trials and non-randomized experimental before-and-after studies, case series, and prospective observational studies of neurotypically developing people up to 18 years old. Studies with people with disabilities and neurodevelopmental disorders were excluded. Interventions researched: horse riding and animal-assisted therapy.

**Data analysis:**

Two judges identified primary studies independently by reading the titles and abstracts, considering the inclusion criteria; a third judge was consulted to resolve divergences. The risk of bias was assessed using the ROBINS-I and ROBINS 2 tools.

**Results:**

Altogether, 131 studies were obtained, and duplicates (27) were removed. Subsequently, 104 studies were analyzed and 77 were excluded. Of the 27 studies evaluated in full text, 21 were excluded. Six studies were eligible for this review – four non-randomized clinical studies and two randomized clinical trials.

**Conclusion:**

The non-randomized studies showed significant improvements in cognitive functions and behavioral and emotional aspects. The randomized studies, on the other hand, found significant gains in social competence.

## INTRODUCTION

Sports and physical activities in general express the Brazilian people's polysemic, multicultural, and mixed-race identity^(1)^. In the case of horse riding, there are four branches in Brazil: equine-assisted therapy (EAT), recreational horse riding, classical equestrianism, and rural equestrianism^(1)^. In the 19th century, horses were already used in high jump and long jump competitions to test them for hunting. It was only in 1900, in Paris, that equestrianism was considered an Olympic sport. This is the only Olympic sport where men and women compete together on equal terms for medals in mixed events^(2)^.

EAT uses horses in an interdisciplinary approach encompassing health, education, and horse riding, seeking the biopsychosocial development of people with disabilities^(3)^. The three-dimensional movement generated by the horse's step is similar to human gait, moving the rider up and down, side to side and forward and backward^(4,5)^. This movement activates the central and autonomic nervous system^(6,7)^.

EAT studies with children and adolescents with disabilities have found gains in cognitive and executive functions^(8)^ and behavioral gains in child autonomy, self-efficacy, and decreased anxiety^(9,10)^. The literature discusses the benefits of riding with people with disabilities, but few studies investigate the effects of horseback riding on the neurotypical population. This review aimed to investigate evidence of horseback riding in the development of language, cognition, and social, emotional, and behavioral aspects in neurotypical children and adolescents, which may contribute to indicating this sport in healthcare focused on prevention and promotion.

## METHODS

The study’s research question is, “How effective is horse riding in the development of language, cognition, and social, emotional, and behavioral aspects in neurotypical children and adolescents?”. The search strategy was based on the PCC strategy (Participants, Concept, Context), namely:

Participants: typically developing children and adolescents up to 18 years old.

Concept: horse riding/EAT/animal-assisted therapy.

Context: development of language, cognitive functions, and behavioral, emotional, and social aspects.

### Search strategy

Searches were conducted in the databases of LILACS, MEDLINE, Web of Science, EMBASE, Scopus, and grey literature, with no date or language restrictions. This review was registered in the Open Science Framework (OSF), under number: DOI 10.17605/OSF.IO/32ETZ

The search strategies used the descriptors related to “Equine-Assisted Therapy”, “Child or Adolescent Development”, Cognition, Socialization, “Language”, and “Child Behavior” (detailed searches are presented in Chart 1).

**Chart 1:** Database search strategies

Database	Search strategy
***LILACS***	((“Terapia Assistida por Equinos” OR hipoterapia OR “Terapia Assistida por Cavalos” OR “Equine-Assisted Therapy” OR “Terapía Asistida por Caballos” OR équithérapie OR equoterapia OR equitação)) AND ((“Desenvolvimento da Linguagem” OR “Language Development” OR “Desarrollo del Lenguaje” OR “Développement du langage oral” OR “Desenvolvimento Infantil” OR “Child Development” OR “Desarrollo Infantil” OR “Développement de l'enfant” OR “Desenvolvimento da Criança” OR “Desenvolvimento do Adolescente” OR “Adolescent Development” OR “Desarrollo del Adolescente” OR “Développement de l'adolescent” OR cognição OR cognition OR cognición OR socialização OR socialization OR socialización OR socialisation OR “Angústia Psicológica” OR “Psychological Distress” OR “Distrés Psicológico” OR “Détresse psychologique” OR “Estresse Emocional” OR “Comportamento Infantil” OR “Child Behavior” OR “Conducta Infantil” OR “Comportement de l'enfant” OR “Comportamento do Adolescente” OR “Adolescent Behavior” OR “Conducta del Adolescente” OR “Comportement de l'adolescent” OR “Adaptação Psicológica” OR “Adaptation, Psychological” OR “Adaptación Psicológica” OR “Adaptation psychologique” OR “Comportamento Adaptativo”)) AND (db:(“LILACS” OR “IBECS” OR “INDEXPSI” OR “BRISA” OR “WPRIM” OR “tese”))
***MEDLINE***	(“Equine-Assisted Therapy”) AND (“Language Development” OR “Child Development” OR “Adolescent Development” OR Cognition OR Socialization OR “Psychological Distress” OR “Child Behavior” OR “Adolescent Behavior” OR “Adaptation, Psychological”)
***EMBASE***	(“hippotherapy”) AND (“Language Development” OR “Child Development” OR “Adolescent Development” OR Cognition OR Socialization OR “distress syndrome” OR “Child Behavior” OR “Adolescent Behavior” OR “psychological adjustment”)
***Scopus and Web of Science***	(“Equine-Assisted Therapy”) AND (“Language Development” OR “Child Development” OR “Adolescent Development” OR Cognition OR Socialization OR “Psychological Distress” OR “Child Behavior” OR “Adolescent Behavior” OR “Adaptation, Psychological”).

The last literature search was on November 2, 2023. Three researchers carried out this study, two of whom received via email the articles for analysis, searched by the librarian of the Federal University of Minas Gerais, and the third researcher was recruited to resolve in the event of discrepancies. After analyzing the titles and abstracts of the first articles consulted by the librarian and selecting the studies for full reading, these articles’ bibliographic references were searched to select studies (according to the inclusion criteria) for abstract and full-text reading.

This review considered the following study designs: randomized clinical trials, non-randomized experimental before-and-after studies, case series, and prospective observational studies with neurotypically developing children (0 to 12 years old) and adolescents (up to 18 years old).

The inclusion criteria were studies addressing the effects of horse riding and EAT on children’s and adolescents’ development of language, cognitive functions, and behavioral, emotional, and social aspects in comparison with a control group (no intervention, waiting list, placebo, or sham) or another intervention (any other active intervention). The outcomes of interest were the development of language, cognition, and behavioral, emotional, and social aspects. The exclusion criteria were studies with people with disabilities or any neurodevelopmental disorders. The review considered interventions with horse riding and animal-assisted therapy.

### Study selection

References were initially identified and exported to the Mendeley bibliographic reference manager, and duplicates were removed. Then, two independent reviewers screened all titles and abstracts and selected potential full texts. Studies that met the inclusion criteria were included in the review. A third reviewer resolved discrepancies between reviewers in two studies.

### Data extraction and analysis

After searching the databases, two judges identified primary studies independently by reading the titles and abstracts, considering the inclusion criteria. A third judge was consulted in case of discrepancies.

The articles selected in the title and abstract screening were read in full to extract data on the sample (number of participants, age range, sex), intervention type (horse riding and animal-assisted therapy), study location, outcomes investigated, follow-ups, and test types. The following additional data were identified: research type, year of publication, nationality of research, journal, and impact factor. The interest group results were extracted regarding short-term (up to 1 month) and long-term (1 year) effects. Short-term follow-up was defined as reassessment immediately after or up to 1 month after the intervention. Only one study followed up and reassessed participants in the long term.

**Figure 1:**
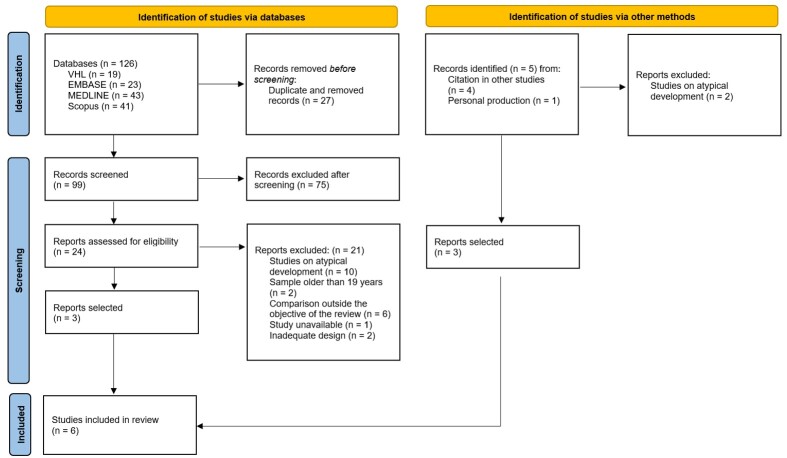
Scoping review preparation steps

A flowchart presenting the steps for preparing this review, according to the Preferred Reporting Items for Systematic Reviews and Meta-Analyses (PRISMA-ScR)^(11)^, is shown in Figure 1.

### Assessment of Methodological Quality

Two researchers assessed the articles; in cases of diverging opinions, the third researcher resolved. They extracted data from each study included in the review and assessed the risk of bias. The latter used two tools developed by the Cochrane Bias Methods Group and the Cochrane Non-Randomized Studies Methods Group, namely: ROBINS-I^(12)^, which assesses the risk of bias in non-randomized intervention studies; and ROBINS-2^(13)^, which assesses the risk of bias in randomized intervention studies. ROBINS-I assesses bias due to confounding, bias due to selection of participants, bias in classification of interventions, bias due to deviations from intended interventions, bias due to missing data, bias in measurement of outcomes, and bias in selection of the reported result. The response options for the signaling questions are yes, probably yes, probably no, no, and no information. From these responses, researchers can judge whether there is a low, moderate, serious, or critical risk of bias or no information regarding that domain. ROBINS-2 assesses bias arising from the randomization process, bias due to deviations from intended interventions, bias due to missing outcome data, bias in the measurement of the outcome, and bias in selection of the reported result. The response options for the signaling questions are yes, probably yes, probably no, no, and no information. From these responses, researchers can judge whether there is a low risk of bias, some concerns, or a high risk of bias.

## RESULTS

### Characteristics of the studies included in the review

The researchers found 126 articles through the search strategy and five from other sources. They excluded 27 duplicates and then analyzed the titles and abstracts of 104 studies, excluding 77. They evaluated the 27 potential full-text studies and excluded 21. The main reasons for excluding them were atypically developing children and adolescents, sample aged over 18 years, comparison outside the objective of the review, and case study design. Six studies were eligible for this review – four non-randomized clinical studies and two randomized clinical trials. It was not possible to perform the meta-analysis due to the insufficient number of clinical trials analyzing the same outcomes.

The description of the studies included in the review is presented in Chart 2.

**Chart 2:** Description of studies included in the review

Study/Country	Journal/Impact factor	Design	Participants	Intervention	Tests and outcomes	Results
Silva et al.^(14)^	Revista de Psicopedagogia	Quasi-experimental	N = 70	12 months of riding, an average of 69.24 lessons in this period, 2x per week, lessons lasting 2:30.	Cognitive and executive functions assessed by NEUPSILIN^(15)^ and NEUPSILIN-Inf^(16)^	Significant improvement (p < 0.05) in the neuropsychological evaluation when comparing the normative values ​​before and after riding lessons, in the tasks of orientation (38.6%, 55.7%), memory (48.6%, 67.1%), oral language (40.0%, 61.4%), motor skills (62.9%, 77.1%), and verbal fluency (47.1%, 65.7%), respectively.
Brazil	IF = 0.049		(males: 32; females: 38)			
			Vulnerable Brazilian children, aged 7 to 17			
Norwood et al.^(17)^	The Journal of Alternative and Complementary Medicine	Quasi-experimental	N = 50	Pre- and post-intervention evaluation of the same group.	Behavioral Rating Inventory of Executive Function (BRIEF)^(18)^ and the Strengths and Difficulties Questionnaire (SDQ)^(19)^, answered by teachers.	BRIEF:
Australia	IF = 2.381		(females: 20; males: 29; other: 1)	5 weeks of horse riding, lasting 2 hours, without a therapist or specific therapeutic program.		Emotional control subscale: no statistically significant difference (z = 469, p = 0.076), but 25 scores improved in the reassessment.
			Aged 12 to 17 years	Control group = 9 subjects from the sample performed the same tests 6 weeks before starting the program. Hence, they were their own controls.		Inhibitory Control subscale:
			Mean age = 13.88 years			Decrease in inattention symptoms after intervention.
			Vulnerable residents of Brisbane and suburbs of Queensland, Australia.			Improvement in Working Memory: (-9.875 [95% CI, -19.684 to -.066], p < .05).
						SQD: improvement in all scores, but only the decrease in hyperactivity was statistically significant.
Tsantefski et al.^(20)^	Health and Social Care in the Community	Quasi-experimental	N = 41 children (mean age 10.26 years)	Pre- and post-horse riding program assessment, of the same group	Strengths and Difficulties Questionnaire (SDQ)^(19)^: answered by parents (n = 41) and teachers (n = 31)	Statistically significant decrease in difficult behavior in the reassessment by parents (M = 18.2 to M = 15.2; p = 0.008), not significant when answered by teachers (M = 17.5 to M = 15.4; p = 0.082)
Australia	IF = 2.39		Children of substance-using parents living in Victoria/Australia.	Duration: 12 weeks, 2-hour sessions.		Decrease in emotional problems reported by parents (M = 5.4 to M = 4.3; p = 0.007), symptoms of hyperactivity (M = 5.2 to M = 4.3; p = 0.031). Reported by teachers there was a decrease in hyperactivity (M = 6.0 to M = 5.3; p = 0.037)
Bachi et al.^(21)^	Clinical Child Psychology and Psychiatry	Non-randomized experimental study	N = 29	Exp group = 7 months of equine-assisted therapy, 14 to 26 sessions lasting 50 minutes.	Measures of self-image (Offer self-image questionnaire OSIQ)^(22)^, self-control (Rosenbaum, 1980, Hebrew version)^(23)^, trust (Children’s Interpersonal Trust Scale)^(24)^, and general satisfaction with life (Student’s Life Satisfaction Scale)^(25)^.	Trust: measurement effect (F=0.005, p>0.05), group effect (F=0.993, p>0.05) and interaction between measurement and group (F=1.425, p>0.05) were not significant.
Israel	IF = 2.12		Exp Group = 15	Control group = contact with horses only in agricultural studies and leisure activities.		Self-Control: improvement in both groups in the reassessment, but neither the group effect (F=0.563, p>0.05) nor the interaction between measurement and group (F=0.119, p>0.05) was significant.
			Control Group = 14			Self-Image: measurement effect (F=1.393, p>0.05), group effect (F=0.63, p>0.05), and interaction between measurement and group (F=0.017, p>0.05) were not significant.
			Adolescents 14 to 18 years old with difficulties in following rules, excessive use of physical or verbal violence to solve problems, lack of self-control, etc.			Overall satisfaction with life: a tendency to increase in the experimental group and decrease in the control group, but not significant (effect measure F=0.257, p>0.05, group effect F=0.133, p>0.05 and interaction between the measure and group F=0.959, p>0.05)
Pendry et al.^(26)^	Journal Primary Prevention	Randomized Clinical Trial	N = 131	Exp group = 11 weeks of equine-assisted therapy, 90-minute individual and group sessions.	Social competence (Devereux Student Strength Assessment - DESSA, LeBuffe et al., 2009^)(27)^: assessed by parents.	Improvement in social competence after intervention (F=1.112, p<0.001)
USA	IF = 1.963		Exp Group = 53	Control group = waited to ride after 16 weeks.	Positive and negative behavior were assessed by 3 raters in all weekly sessions. Animal Assisted Therapy^(28)—^Psychosocial Session	Positive behavior increased significantly after intervention (p<0.001) and negative behavior decreased (p<0.001).
			Control Group = 60		Form (AAT-PSF, Chandler, 2005)^(28)^.	Attendance at the animal-assisted therapy program is significantly associated with changes in adaptive behavior in the sample (p<0.001).
			North American children in 5^th^ to 8^th^ grades			
Hauge et al.^(29)^	International Journal of Adolescence and Youth	Randomized Clinical Trial	N = 75	4 months of riding, weekly lessons lasting 2 hours.	Social competence (Resilience Scale for Adolescents READ)^(30)^, self-esteem (Global self-worth)^(31)^, general self-efficacy (General Self Efficacy scale for adolescents)^(32)^, and adolescents' experience with horse riding (Questionnaires for the intervention in relation to activities with horses)^(33)^.	Social competence: significant improvement (p<0.05)
Norway	IF = 4.72		(males: 10; females: 65)			Self-esteem and Self-efficacy: a slight increase in averages after the intervention, with no statistical difference compared to the control group
			Exp group = 42			
			Control group = 33			
			Norwegian adolescents aged 12 to 15			

**Caption:** IF = impact factor; N = sample size; Exp = experimental group

Three of them were quasi-experimental studies^(14,17,20)^, one was a non-randomized experimental study^(21)^, and the other two were randomized clinical trials^(26,29)^. The intervention time ranged from 1 to 12 months, with weekly classes lasting 50 to 150 minutes. The sample size of the experimental groups ranged from 15 to 70 participants. Four studies^(14,17,20,29)^ used horse riding interventions, and two studies^(21,26)^ used EAT. The studies were published between 2012 and 2023, five of them in English, and one in Portuguese. The countries where the studies were conducted were also diverse, being one in Brazil, two in Australia, one in Israel, one in the United States, and one in Norway. The study population’s age ranged from 7 to 17 years for both sexes. The participants of four studies were socially vulnerable or at risk for behavioral situations^(14,17,20,21)^; two studies did not mention this information^(26,29)^.

The outcomes were diverse. Two studies (33.3%) investigated cognitive functions, language, and executive functions^(14,17)^; three studies (50%) discussed behavioral aspects^(17,20,26)^; two studies (33.3%) reported emotional aspects^(21,29)^; and another two studies (33.3%) evaluated social aspects^(26,29)^. The tests used in each study and the outcomes they analyzed are described in Chart 3.

**Chart 3:** Outcomes analyzed and tests applied in the scoping reviews

Outcomes analyzed	Tests applied
Emotions and behavior^(17,20)^	Strengths and Difficulties Questionnaire (SDQ)^(19)^
Cognitive functions^(14)^, language^(14)^, and executive functions^(14,17)^	NEUPSILIN^(15)^, NEUPSILIN-Inf^(16)^
	Behavioral Rating Inventory of Executive Function (BRIEF)^(18)^
Self-image^(21)^	Offer self-image questionnaire OSIQ^(22)^
Self-control^(21)^	Rosenbaum, 1980, Hebrew version^(23)^
Trust^(21)^	Children’s Interpersonal Trust Scale^(24)^
Overall satisfaction with life^(21)^	Student’s Life Satisfaction Scale^(25)^
Social competence^(26,29)^	Devereux Student Strength Assessment
	(DESSA)^(27)^ Resilience Scale for Adolescents -READ^(30)^
Positive and negative behaviors^(26)^	Animal Assisted Therapy—Psychosocial Session Form^(28)^
Self-esteem^(29)^	Global self-worth^(31)^
Self-efficacy^(29)^	General Self Efficacy scale for adolescents^(32)^
Adolescents' experiences with horse riding^(29)^	Questionnaires for the intervention in relation to activities with horses^(33)^

The ROBINS-I and ROBINS 2 analyses are shown in Charts 4 and 5, respectively.

**Chart 4:** Analysis of the methodological quality of non-randomized studies - ROBINS-I

Study	Bias due to confounding	Bias due to selection of participants	Bias in classification of interventions	Bias due to deviations from intended interventions	Bias due to missing data	Bias in measurement of outcomes	Bias in selection of the reported result	Overall
Silva et al.^(14)^	Moderate	Low	Low	Low	Low	Moderate	Low	Moderate
Norwood et al.^(17)^	Moderate	Low	Low	Low	Low	Moderate	Low	Moderate
Tsantefski et al.^(20)^	Moderate	Low	Low	Low	Low	Moderate	Low	Moderate
Bachi et al.^(21)^	Moderate	Low	Low	Low	Low	Moderate	Low	Moderate

**Caption:** Low: Low risk of bias; Moderate: Moderate risk of bias

**Chart 5:** Analysis of the methodological quality of randomized studies - ROBINS 2

Study	Domains						
	D1a	D1b	D2	D3	D4	D5	Overall
Hauge et al.^(29)^	+	+	+	+	+	+	+
Pendry et al.^(26)^	+	+	+	+	+	!	!

**Caption:** D1a: randomization process; D1b: identification and recruitment of participants; D2: deviations from intended interventions; D3: missing data; D4: measurement of the outcome; Dd5: selection of the reported results. Green boxes with a plus sign: low risk of bias; yellow boxes with an exclamation mark: Some concerns about the risk of bias

This analysis shows that all non-randomized studies had a moderate risk of bias, mainly in the domains of confounding bias and outcome measurement bias. The analysis of the two randomized studies included in the review found that one had a low risk of bias and the other had some concerns regarding the risk of bias because of domain five, due to a failure in the blinding of the study.

## DISCUSSION

This study aimed to investigate evidence of horse riding and EAT in neurotypical children and adolescents. It found that these practices improved some cognitive, language, behavioral, emotional, and social aspects, although the quality of the scientific evidence is low since most of the studies are non-randomized clinical trials (66%). Randomized clinical trials would be the most appropriate to answer questions related to horse riding intervention (i.e., the one capable of providing more reliable information), but these corresponded to only 33% of the studies in this review.

Regarding outcomes found in the review, only the non-randomized study^(14)^ screened several cognitive functions. It investigated arithmetic skills, attention, visual perception, executive functions, oral and written language, orientation, memory, verbal fluency, and praxis – the last five improving significantly after the intervention. Such gains can be justified by the results of research on horseback riding and brain connectivity, which demonstrate a close relationship between the thalamic region bilaterally and the higher functions of attention, language, memory, and executive function^(34)^, as this brain area is possibly activated after horseback riding^(6)^. Moreover, functions such as orientation are complex and may require integration between attention, memory, and perception.

Memory is the ability to acquire, retain, and use knowledge, involving several cortical and subcortical components. Working memory is a limited attention system, storing information briefly, only while a task is being performed. Attention and working memory tasks are interrelated^(16)^. Studies indicate that horseback riding helps activate the frontal lobe and right caudate nucleus, structures responsible for these skills^(6,35)^. The non-randomized study^(17)^ also investigated working memory and found significant improvement in reassessment. However, each study^(14,17)^ used a different test to assess working memory, and the riding time varied – one study lasted 5 weeks^(17)^, and the other lasted 12 months^(14)^. It is known that interventions that extend over longer periods tend to provide more sustainable and significant gains in cognitive skills. They reinforce and consolidate acquired skills, facilitating the transfer of these gains to other areas of life^(36)^.

Oral language was another outcome with significant gains, though investigated in only one study^(14)^. Studies demonstrate the relationship between good oral language performance and reading competence^(37-41)^. This is important since socially vulnerable populations perform poorly in reading^(42)^. Stimulating oral language with horse riding can be a path to acquiring gains in learning, particularly in reading^(43)^.

Regarding executive functions, a non-randomized study^(14)^ investigated problem-solving skills and verbal fluency, with significant improvement only in verbal fluency – an executive process involving controlled production of words based on a semantic or phonological rule in a given time^(16)^. Considering the brain region responsible for verbal fluency^(44)^, an experimental study compared the functional brain connectivity between neurotypical children and children with attention deficit disorder before and after 4 weeks of horseback riding and indicated greater connectivity of the superior frontal gyrus in neurotypical participants^(6)^.

Another non-randomized study^(17)^ investigated inhibitory control (an executive function skill as well). The brain area responsible for executive function is the prefrontal cortex bilaterally, and studies indicate that these skills improve with advancing age^(16,35)^.

Motor skills activate the frontal and parietal lobes, promoting visual discrimination and synthesis, spatial orientation, and other prerequisites to developing writing, drawing, and so on^(14,16)^. Horse riding favors balance, gross and fine motor coordination, and body awareness^(45-47)^.

The non-randomized studies^(17,20)^ reported a statistically significant decrease in hyperactivity, and only one study^(20)^ found a statistically significant decrease in parent-reported emotional problems and a decrease in difficult behaviors. One study performed EAT^(20)^ for 12 weeks, and the other one^(17)^ reinforced in its methodology that the intervention would not include a therapist, only horseback riding and human-horse interaction for 5 weeks. EAT studies with people with attention-deficit/hyperactivity disorder lasting 12 weeks^(48-51)^ indicated significant behavioral gains regarding decreased negative problems, increased positive behaviors, reduced anxiety, social problems, and hyperactivity, and improved quality of life. The importance of human-horse interaction is discussed as the key to curing social and emotional problems, as the person can open up and reveal their feelings without fear of judgment^(50)^.

The two randomized studies^(26,29)^ identified significantly improved social competence after the horse riding intervention, and the results in one of them^(26)^ indicated statistically significantly decreased negative behaviors and increased positive behaviors. Social competence is related to the ability to articulate thoughts, actions, and feelings according to the person’s needs, situation, and culture. This ability has positive consequences for the individual and their relationships with others, being an important factor for social adjustment and success at school^(52)^. This study also discussed that behavioral problems are directly related to children and adolescents’ poor academic performance and reduced repertoire of social skills. EAT studies indicate improved anxiety and general behavior in adolescents at risk^(53-55)^. A randomized study^(56)^ demonstrated a decrease in cortisol levels in adolescents after 11 weeks of horse riding, suggesting that human-horse interaction decreases stress levels and the stress response, being a protective influence against developing psychopathologies and health problems^(56)^.

An important aspect of the findings is the number of studies, as there were more non-randomized than randomized studies. This directly impacts the methodological quality of research, which is important in the process of generating scientific evidence^(57)^. More clinical trials are needed in both horse riding and EAT to guarantee their effectiveness in human development. The methodological diversity of the studies in the review also made it difficult to generalize the findings, as it was not possible to compare them.

However, scientific evidence is based not only on methodological quality but also on professional clinical experience and patient preferences^(58)^. Studies aiming at evidence-based practices are greatly important in clinical practice and science, as they guide healthcare^(58)^ and direct and transform clinical practice. Although the articles found in this review have methodological issues that need to be improved, they were believed to be carried out because clinical expertise had confirmed the social, behavioral, emotional, and cognitive benefits of horse riding to those who practice it.

Systematic reviews and meta-analyses are at the forefront of evidence-based practice. However, an important limitation of this scoping review was the insufficient number of randomized clinical trials investigating the same outcomes to perform this analysis. Five studies in this review had a moderate risk of bias, and one randomized study had a low risk of bias. Therefore, most did not present high quality that could prove the observed effect. Methodological rigor contributes to reduced bias and more reliable results. In this sense, further studies may find different outcomes.

Horse riding is a more elitist sport and somewhat inaccessible to the general population. Hence, research in this area requires funding from development agencies to recruit enough participants for a good randomized clinical trial. The major obstacle for studies with adequate methodological quality may be the lack of partnerships between equestrian centers and universities. Obtaining evidence for horse riding opens a new horizon with perspectives for EAT as well. Systematic reviews with meta-analyses on EAT with children with cerebral palsy^(58)^ and autism spectrum disorder^(59)^ found positive scientific evidence for this therapeutic modality in this population.

Methodological aspects of the studies included in the review stand out as limitations of the present study. For instance, they lacked a control group, did not allocate participants randomly, and had different intervention durations, compromising the quality of their scientific evidence.

## CONCLUSION

The non-randomized articles analyzed in this review reported horse riding benefits in outcomes of cognitive functions, language, and behavioral and emotional aspects. However, the interventions and outcome measurements were not homogeneous, posing a limitation for the conclusions of this study. Moreover, the randomized studies in this review found benefits in social competence after horse riding.
