# Supplementary material for: Benefits of horseback riding for neurotypical children and adolescents: a scoping review
Source: Codas. 2025 Mar 31;37(3):e20240083. doi: 10.1590/2317-1782/e20240083en (PMC11963882; doi:10.1590/2317-1782/e20240083en)
Supplement: Translation

# Translation: Benefícios da equitação em crianças e adolescentes neurotípicos: revisão de escopo

**DOI:** 10.1590/2317-1782/e20240083pt

**Authors:** Flaviana Gomes da Silva, Danielle Diniz de Paula, Luciana Mendonça Alves, Juliana Nunes Santos

**Affiliations:** 1 Departamento da Pós-graduação em Ciências Fonoaudiológicas, Faculdade de Medicina, Universidade Federal de Minas Gerais – UFMG - Belo Horizonte (MG), Brasil.; 2 Instituto de Ciência e Tecnologia – ICTIN, Universidade Federal de Lavras – UFLA - Lavras (MG), Brasil.

**Keywords:** Terapia Assistida por Cavalos, Desenvolvimento da Criança e Adolescente, Cognição, Socialização, Comportamento Infantil, Linguagem

## Abstract

**Objetivo:**

investigar evidências da equitação no desenvolvimento de linguagem, cognição, aspectos sociais, emocionais e comportamentais em crianças e adolescentes neurotípicos.

**Estratégia de pesquisa:**

Consulta nas bases de dados da Lilacs, MEDLINE, *Web of Science*, EMBASE, *Scopus* e literatura cinzenta, sem restrições de data ou idioma. Registro na plataforma Open Science Framework (OSF), sob número: DOI 10.17605/OSF.IO/32ETZ. Para estratégias de busca: “*Equine-Assisted Therapy*”, “*Child or Adolescent Development*", *Cognition, Socialization* e "*Child Behavior*”.

**Critérios de seleção:**

Ensaios clínicos randomizados e estudos experimentais não randomizados de antes e depois, série de casos e observacionais prospectivos de pessoas com desenvolvimento neurotípico com até 18 anos de idade. Excluídos os estudos com pessoas com deficiência e transtornos do neurodesenvolvimento.

**Análise dos dados:**

Equitação e terapia assistida por animais. Análise dos dados: A identificação de estudos primários foi realizada por dois juízes de forma independente, por meio de leitura dos títulos e resumos baseada nos critérios de inclusão e um terceiro juiz era consultado para caso de desempate. O risco de viés foi avaliado pelas ferramentas ROBINS-I e a ROBINS-2.

**Resultados:**

Foram obtidos 131 estudos, sendo removidos os duplicados (27). Em seguida, 104 estudos foram analisados e 77 excluídos. Dos 27 estudos avaliados integralmente, 21 foram excluídos. Seis estudos foram elegíveis para esta revisão, sendo quatro estudos clínicos não randomizados e dois ensaios clínicos randomizados.

**Conclusão:**

Os estudos não randomizados apontaram melhoras significativas quanto às funções cognitivas, aspectos comportamentais e emocionais. Já os estudos randomizados, encontraram ganhos significativos quanto à competência social.

## INTRODUÇÃO

O esporte no Brasil, bem como as atividades físicas no geral, expressam a identidade polissêmica, multicultural e miscigenada de seu povo^(1)^. No caso da equitação, há quatro vertentes no Brasil: a equoterapia, a equitação de lazer, o hipismo clássico e o rural^(1)^. No século 19 o cavalo já era utilizado em competições de salto em altura e salto em distância, como forma de testá-los para a caça. Somente no ano de 1900, em Paris, que o hipismo foi considerado um esporte olímpico. Esse é o único esporte olímpico que homens e mulheres competem juntos disputando medalhas, em provas mistas, em condição de igualdade^(2)^.

A equoterapia é um método terapêutico que utiliza o cavalo dentro de uma abordagem interdisciplinar nas áreas de saúde, educação e equitação, buscando o desenvolvimento biopsicossocial de pessoas com deficiência^(3)^. O movimento tridimensional gerado pelo passo do cavalo é semelhante à marcha humana, deslocando o cavaleiro para cima e para baixo, para um lado e para o outro e para frente e para trás^(4,5)^. Esse movimento leva à ativação do sistema nervoso central e autônomo^(6,7)^.

Estudos sobre equoterapia com crianças e adolescentes com deficiência constataram ganhos nas funções cognitivas e executivas^(8)^, além dos ganhos comportamentais de autonomia infantil, autoeficácia e diminuição da ansiedade^(9,10)^. São discutidos na literatura os benefícios da montaria com pessoas com deficiência e poucos estudos investigam os efeitos da equitação na população neurotípica. O objetivo desta revisão é investigar evidências da equitação no desenvolvimento de linguagem, cognição, aspectos sociais, emocionais e comportamentais em crianças e adolescentes neurotípicos, podendo contribuir para a indicação deste esporte no cuidado em saúde, com foco na prevenção e promoção.

## MÉTODO

A pergunta norteadora do estudo foi: Qual a efetividade da equitação no desenvolvimento de linguagem, cognição, aspectos sociais, emocionais, comportamentais em crianças e adolescentes neurotípicos? A estratégia de busca foi baseada na estratégia PCC (Participantes, Conceito, Contexto), a saber:

Participantes: crianças e adolescentes com desenvolvimento típico até 18 anos

Conceito: equitação/equoterapia/terapia assistida por animais

Contexto: desenvolvimento de linguagem, funções cognitivas, aspectos comportamentais, emocionais e sociais.

### Estratégia de pesquisa

Foram realizadas buscas nas bases de dados da Lilacs, MEDLINE, *Web of Science*, EMBASE, *Scopus* e literatura cinzenta, sem restrições de data ou idioma. Esta revisão foi registrada na plataforma Open Science Framework (OSF), sob número: DOI 10.17605/OSF.IO/32ETZ

Para estratégias de busca, foram utilizados os descritores relacionados a “*Equine-Assisted Therapy*”, “*Child or Adolescent Development*", *Cognition, Socialization, “Language”* e "*Child Behavior*” (as buscas detalhadas estão apresentadas no Quadro 1).

**Quadro 1:** Estratégias de busca nas bases de dados

Base de dados	Estratégia de busca
***LILACS***	(("Terapia Assistida por Equinos" OR hipoterapia OR "Terapia Assistida por Cavalos" OR "Equine-Assisted Therapy" OR "Terapía Asistida por Caballos" OR équithérapie OR equoterapia OR equitação)) AND (("Desenvolvimento da Linguagem" OR "Language Development" OR "Desarrollo del Lenguaje" OR "Développement du langage oral" OR "Desenvolvimento Infantil" OR "Child Development" OR "Desarrollo Infantil" OR "Développement de l'enfant" OR "Desenvolvimento da Criança" OR "Desenvolvimento do Adolescente" OR "Adolescent Development" OR "Desarrollo del Adolescente" OR "Développement de l'adolescent" OR cognição OR cognition OR cognición OR socialização OR socialization OR socialización OR socialisation OR "Angústia Psicológica" OR "Psychological Distress" OR "Distrés Psicológico" OR "Détresse psychologique" OR "Estresse Emocional" OR "Comportamento Infantil" OR "Child Behavior" OR "Conducta Infantil" OR "Comportement de l'enfant" OR "Comportamento do Adolescente" OR "Adolescent Behavior" OR "Conducta del Adolescente" OR "Comportement de l'adolescent" OR "Adaptação Psicológica" OR "Adaptation, Psychological" OR "Adaptación Psicológica" OR "Adaptation psychologique" OR "Comportamento Adaptativo")) AND ( db:("LILACS" OR "IBECS" OR "INDEXPSI" OR "BRISA" OR "WPRIM" OR "tese"))
***MEDLINE***	("Equine-Assisted Therapy") AND ("Language Development" OR "Child Development" OR "Adolescent Development" OR Cognition OR Socialization OR "Psychological Distress" OR "Child Behavior" OR "Adolescent Behavior" OR "Adaptation, Psychological")
***EMBASE***	("hippotherapy") AND ("Language Development" OR "Child Development" OR "Adolescent Development" OR Cognition OR Socialization OR "distress syndrome" OR "Child Behavior" OR "Adolescent Behavior" OR "psychological adjustment")
***Scopus e Web of Science***	("Equine-Assisted Therapy") AND ("Language Development" OR "Child Development" OR "Adolescent Development" OR Cognition OR Socialization OR "Psychological Distress" OR "Child Behavior" OR "Adolescent Behavior" OR "Adaptation, Psychological").

A data da última busca de literatura utilizada foi em dois de novembro de 2023. Ao todo foram três pesquisadores envolvidos com a realização desse estudo, sendo dois pesquisadores que receberam os artigos para análise, pesquisados pela bibliotecária da Universidade Federal de Minas Gerais, via email, e o terceiro pesquisador era recrutado como critério de desempate em alguma divergência. Após a análise de títulos e resumos dos primeiros artigos consultados pela bibliotecária e seleção dos estudos para leitura completa, foi feita a busca nas próprias referências bibliográficas desses artigos e foram selecionados estudos (de acordo com os critérios de inclusão) para leitura dos resumos e texto completo.

Para a presente revisão foram considerados os seguintes desenhos de estudo: ensaios clínicos randomizados e estudos experimentais não randomizados de antes e depois, série de casos e observacionais prospectivos de crianças (zero a 12 anos) e adolescentes (até 18 anos de idade) com desenvolvimento neurotípico.

Os critérios de inclusão adotados foram aqueles que compararam o efeito da equitação e equoterapia no desenvolvimento de crianças e adolescentes quanto ao desenvolvimento de linguagem, ganhos comportamentais, funções cognitivas, aspectos emocionais e sociais, ao grupo controle (sem intervenção, lista de espera, placebo ou *SHAM*) ou outra intervenção (qualquer outra intervenção ativa). Os desfechos de interesse foram desenvolvimento da linguagem, cognição, comportamento, aspectos emocionais e aspectos sociais. Os de exclusão foram os estudos com pessoas com deficiência ou quaisquer transtornos do neurodesenvolvimento. Para as intervenções foram consideradas a equitação e terapia assistida por animais.

### Seleção dos estudos

Inicialmente, as referências identificadas foram exportadas para o gerenciador de referências bibliográficas *Mendeley* e as duplicatas foram removidas. Em seguida, dois revisores independentes examinaram todos os títulos e resumos e selecionaram textos completos em potencial. Os estudos que preencheram os critérios de inclusão foram incluídos na revisão. Um terceiro revisor resolveu as discrepâncias entre os revisores em dois estudos.

### Extração e análise dos dados

Após a busca nas bases de dados, a identificação de estudos primários foi realizada por dois juízes de forma independente, por meio de leitura dos títulos e resumos, baseada nos critérios de inclusão e um terceiro juiz era consultado para caso de desempate.

Os artigos selecionados na triagem de títulos e resumos foram lidos na íntegra para extração de dados relacionados às seguintes categorias: casuística (número de participantes, faixa etária, gênero), tipo de intervenção (equitação e terapia assistida por animais), local de realização do estudo, desfechos investigados, *follow ups* e tipos de testes utilizados. Como dados complementares identificaram-se: tipo de pesquisa, ano de publicação, nacionalidade das pesquisas, periódico e fator de impacto. Os resultados para os grupos de interesse foram extraídos para efeito de curto (até um mês) e longo prazo (um ano). O seguimento de curto prazo foi definido pela reavaliação imediatamente após ou até um mês após a intervenção. Apenas um estudo realizou o acompanhamento e reavaliação dos participantes em longo prazo.

O fluxograma realizado de acordo com o *Preferred Reporting Items for Systematic Reviews and Meta-Analyses* (PRISMA- ScR)^(11)^, das etapas para elaboração da presente revisão está apresentado na Figura 1.

**Figura 1:** Etapas de elaboração da revisão de escopo

### Avaliação da qualidade metodológica

Os artigos foram avaliados por dois pesquisadores e nos casos de divergências de opiniões, o terceiro pesquisador realizou o desempate. Para cada estudo incluído, os dados foram extraídos e o risco de viés foi avaliado. Para essa análise do risco de viés, foram utilizadas duas ferramentas desenvolvidas por membros da *Cochrane Bias Methods Group*e da*Cochrane Non-Randomised Studies Methods Group*, sendo a ROBINS-I^(12)^ que avalia o risco de viés em estudos de intervenção não randomizados e a ROBINS-2^(13)^ que avalia o risco de viés em estudos de intervenção randomizados. Os domínios avaliados pela ROBINS-I são: viés de confusão, de seleção dos participantes do estudo, de classificação da intervenção, desvios nas intervenções pretendidas, devido a dados faltantes, na medição dos resultados e na seleção do resultado reportado. As opções de resposta para as questões de sinalização são: sim, provavelmente sim, provavelmente não, não e sem informação. A partir dessas respostas os pesquisadores podem julgar se tem baixo risco de viés, moderado risco, sério risco, risco crítico e sem informação a respeito daquele domínio. Os domínios avaliados pela ROBINS-2 são: alocação aleatória, desvios nas intervenções pretendidas, dados faltantes, medidas dos resultados e seleção dos resultados reportados. As opções de resposta para as questões de sinalização são: sim, provavelmente sim, provavelmente não, não e sem informação. A partir dessas respostas os pesquisadores podem julgar se tem baixo risco de viés, algumas preocupações e alto risco de viés.

## RESULTADOS

### Características dos estudos incluídos

Um total de 126 artigos foram identificados na estratégia de busca e cinco em outras fontes. Foram excluídos 27 artigos duplicados. Depois de remover duplicatas, títulos e resumos de 104 estudos foram analisados e 77 foram excluídos. Os 27 estudos completos em potencial foram avaliados e 21 foram excluídos. Os principais motivos da exclusão dos estudos foram: criança e adolescente com desenvolvimento atípico, amostra com idade superior a 18 anos, comparação fora do objetivo da revisão, e por fim, delineamento do tipo estudo de caso. Seis estudos foram elegíveis para esta revisão, sendo quatro estudos clínicos não randomizados e dois ensaios clínicos randomizados. Não foi possível realizar a metanálise, devido ao número insuficiente de ensaios clínicos com os mesmos desfechos analisados.

A descrição dos estudos incluídos na revisão está apresentada no Quadro 2.

**Quadro 2:** Descrição dos estudos incluídos na revisão

**Estudo/País**	**Periódico/Fator de Impacto**	**Delineamento**	**Participantes**	**Intervenção**	**Testes e desfechos**	**Resultados**
Silva et al.^(14)^	Revista de Psicopedagogia	Quase experimental	N=70	12 meses de equitação, média de 69,24 aulas nesse período, 2x por semana, aulas de 2h30min de duração.	Funções Cognitivas e Executivas avaliadas pelo NEUPSILIN^(15)^ e NEUPSILIN-Inf^(16)^	Melhora significativa (p<0,05) na avaliação neuropsicológica ao comparar os valores normativos antes e após aulas de equitação, nas tarefas de orientação (38,6%, 55,7%), memória (48,6% e 67,1%), linguagem oral (40,0% e 61,4%) praxias (62,9% e 77,1%) e fluência verbal (47,1% e 65,7%) respectivamente.
Brasil	FI= 0,049		(masc:32 fem: 38)			
			Crianças brasileiras em situação de vulnerabilidade, entre 7 a 17 anos			
Norwood et al.^(17)^	The Journal of Alternative and Complementary Medicine	Quase experimental	N= 50	Avaliação pré e pós intervenção de um mesmo grupo.	Inventário de Avaliação Comportamental de Função Executiva - *Behavioral Rating Inventory of Executive Function* (BRIEF)^(18)^ e o Strengths *and Difficulties Questionnaire* (SDQ)^(19)^: preenchido pelos professores.	BRIEF:
Austrália	FI= 2.381		(fem: 20/masc:29/	5 semanas de equitação, com duração de 2 horas, sem a presença de terapeuta ou programa terapêutico específico.		Subescala de controle emocional: não apresentou diferença estatisticamente significativa (z= 469, p = 0.076), porém 25 escores melhoraram na reavaliação.
			outro: 01)	Grupo controle= 9 sujeitos da amostra realizaram os mesmos testes 6 semanas antes de iniciar o programa, permitindo que eles atuassem como seus próprios controles.		Subescala de Controle Inibitório:
			Idade entre 12 e 17 anos			Diminuição da sintomatologia de desatenção pós intervenção.
			média de idade = 13,88 anos			melhora da Memória de Trabalho: (-9.875 (95% CI, -19.684 para -.066), p < .05).
			Situação de vulnerabilidade			
			Residentes de Brisbane e Subúrbios de Queensland, Austrália.			SQD: melhora em todos os escores, porém somente a diminuição da hiperatividade que foi significativo estatisticamente.
Tsantefski et al.^(20)^	Health and Social Care in the Community	Quase experimental	N= 41 crianças (média de 10,26 anos)	Avaliação pré e pós programa de equitação, do mesmo grupo	Strengths *and Difficulties Questionnaire* (SDQ)^(19)^: preenchido pelos pais (n=41) e professores (n=31)	Diminuição estatisticamente significativa do comportamento difícil na reavaliação pelos pais (M=18,2 para M=15,2; p=0,008) e não foi significativo quando respondido pelos professores (M=17,5 para M=15,4; p= 0,082)
Austrália	FI= 2.39		Crianças residentes em Vitória/Austrália, filhos de pais usuários de substâncias.	Duração: 12 semanas e sessões com 2 horas de duração.		Diminuição nos problemas emocionais relatados pelos pais (M=5,4 para M=4,3; p=0,007), sintomas de hiperatividade (M=5,2 para M=4,3; p=0,031). Relatado pelos professores houve diminuição da hiperatividade (M=6,0 para M= 5,3; p= 0,037)
Bachi et al.^(21)^	Clinical CHild Psychology and Psychiatry	Experimental não- randomizado	N= 29	Grupo exp= 7 meses de sessões de equoterapia, 14 a 26 sessões com duração de 50 minutos.	Medidas de autoimagem (*Offer self-image questionnaire OSIQ)* ^(22)^, autocontrole (Rosenbaum, 1980, versão hebraica^(23)^, confiança (*Children’s Interpersonal Trust Scale)* ^(24)^ e satisfação geral com a vida (*Student’s Life Satisfaction Scale)* ^(25)^.	Confiança: efeito da medição (F=0,005, p>0,05), o efeito do grupo (F=0,993, p>0,05) e a interação entre a medida e o grupo (F=1,425, p>0,05), não foram significativas.
Israel	FI= 2.12		Grupo exp= 15	Grupo Controle= contato com o cavalo somente nos estudos agrícolas e atividades de lazer.		Autocontrole: melhora nos dois grupos, na reavaliação, porém nem o efeito do grupo (F=0,563, p> 0,05), nem a interação entre a medida e o grupo (F=0,119, p>0,05) foram significativos.
			Grupo Cont= 14			Autoimagem: efeito da medida (F=1,393, p>0,05), efeito do grupo (F=0,63, p>0,05) e interação entre a medida e o grupo (F=0,017, p>0,05), não foram significativos.
			Adolescentes entre 14 a 18 anos com dificuldades em seguir regras, uso excessivo de violência física ou verbal para resolver problemas, falta de autocontrole etc.			Satisfação geral com a vida: tendência de aumento no grupo experimental e diminuição no grupo controle, porém não significativo (medida de efeito F=0,257, p>0,05, efeito do grupo F=0,133, p> 0,05 e interação entre a medida e grupo F=0,959, p>0,05)
Pendry et al.^(26)^	Journal Primary Prevention	Ensaio Clínico Randomizado	N= 131	Exp= 11 semanas de equoterapia, sessões de 90 minutos individuais e em grupo.	Competência social (Devereux Student Strength Assessment - DESSA, LeBuffe et al., 2009)^(27)^: avaliado pelos pais.	Melhora da competência social após intervenção (F=1,112, p<0,001)
EUA	FI= 1.963		Grupo exp = 53	Controle= esperou para montar após 16 semanas.	Comportamento positivo e negativo foi julgado por 3 avaliadores em todas as sessões semanais Animal Assisted^(28)^ Therapy—Psychosocial Session	Comportamento positivo aumentou significativamente após intervenção (p< 0,001) e comportamento negativo diminuiu (p<0,001).
			Grupo Cont= 60		Form (AAT-PSF, Chandler, 2005)^(28)^.	Frequência no programa de terapia assistida por animais está significativamente associado à mudança no comportamento adaptativo da amostra (p<0,001).
			Crianças norte americanas do 5º ao 8º ano			
Hauge et al.^(29)^	International Journal of Adolescence and Youth	Ensaio Clínico Randomizado	N= 75	4 meses de equitação, aulas semanais e com duração de 2h.	Competência social (*Resilience Scale for Adolescents READ)* ^(30)^, autoestima (*Global self-worth)* ^(31)^ e autoeficácia geral (*General Self Efficacy scale for adolescents)* ^(32)^ e experiência dos adolescentes com a equitação (*Questionnaires for the intervention in relation to activities with horses)* ^(33)^.	Competência social: melhora significativa (p< 0,05)
Noruega	FI= 4,72		(masc: 10/ fem: 65)			Autoestima e Autoeficácia: apresentaram discreto aumento das médias após intervenção, sem diferença estatística com o grupo controle
			Grupo Exp= 42			
			Grupo cont = 33			
			Adolescentes noruegueses de 12 a 15 anos			

**Legenda:** FI= fator de impacto; N = tamanho da amostra; fem= gênero feminino; mas= gênero masculino; Exp = grupo experimental; Cont = grupo controle

Conforme observado entre os artigos incluídos, três deles apresentaram o delineamento quase experimental^(14,17,20)^, um experimental não randomizado^(21)^ e os outros dois foram ensaios clínicos randomizados^(26,29)^. O tempo de intervenção variou de um a 12 meses, com aulas semanais e a duração de cada aula variou entre 50 minutos e 150 minutos de duração. O tamanho da amostra do grupo experimental variou de 15 a 70 participantes. Quanto à intervenção, quatro estudos^(14,17,20,29)^ mencionaram a prática da equitação e dois estudos^(21,26)^ descreveram a equoterapia. Dentre os estudos incluídos na revisão, cinco foram publicados em inglês e um em português e os anos de publicação foram entre 2012 a 2023. Os países que foram realizadas as pesquisas também foram diversificados, sendo um no Brasil, dois na Austrália, um em Israel, um nos Estados Unidos e outro na Noruega. A idade da população estudada variou entre sete e dezessete anos de ambos sexos. Em quatro estudos os participantes estavam em contexto de vulnerabilidade social ou em risco para situações comportamentais^(14,17,20,21)^ e dois estudos não mencionaram essa informação^(26,29)^.

Os desfechos foram diversificados, com dois estudos (33,3%) investigando as funções cognitivas, linguagem e funções executivas^(14,17)^ , três estudos (50%) discutiram aspectos comportamentais^(17,20,26)^, dois (33,3%) relataram sobre aspectos emocionais^(21,29)^ e outros dois estudos (33,3%) avaliaram os aspectos de competência social^(26,29)^. Os testes utilizados em cada estudo e os desfechos analisados, estão descritos no Quadro 3.

**Quadro 3:** Desfechos analisados e testes aplicados nos estudos da revisão de escopo

Desfechos Analisados	Testes utilizados
Emoções e Comportamento^(17,20)^	Strengths *and Difficulties Questionnaire* (SDQ)^(19)^
Funções Cognitivas^(14)^, linguagem^(14)^ e funções executivas^(14,17)^	NEUPSILIN^(15)^ , NEUPSILIN-Inf^(16)^
	*Behavioral Rating Inventory of Executive Function (BRIEF)* ^(18)^
Autoimagem^(21)^	*Offer self-image questionnaire OSIQ* ^(22)^
Autocontrole^(21)^	Rosenbaum, 1980, versão hebraica^(23)^
Confiança^(21)^	*Children’s Interpersonal Trust Scale* ^(24)^
Satisfação geral com a vida^(21)^	*Student’s Life Satisfaction Scale* ^(25)^
Competência Social^(26,29)^	*Devereux Student Strength Assessment* (DESSA)^(27)^
	*Resilience Scale for Adolescents -READ* ^(30)^
Comportamentos positivos e negativos^(26)^	*Animal Assisted Therapy—Psychosocial Session Form* ^(28)^
Autoestima ^(29)^	*Global self-worth* ^(31)^
Auto eficácia^(29)^	*General Self Efficacy scale for adolescents* ^(32)^
Experiências dos adolescentes com a a equitação^(29)^	Q*uestionnaires for the intervention in relation to activities with horses* ^(33)^

As análises ROBINS-I e ROBINS 2 podem ser visualizadas no Quadro 4 e Quadro 5, respectivamente.

**Quadro 4:** Análise da qualidade metodológica dos estudos não randomizados - ROBINS-I

Estudo	Viés de Confundimento	Viés de Seleção dos participantes	Viés de Classificação das intervenções	Viés de desvios da intervenção pretendida	Viés de perda de dados	Viés de medida dos desfechos	Viés de seleção dos resultados reportados	Geral
Silva et al.^(14)^	Moderado	Baixo	Baixo	Baixo	Baixo	Moderado	Baixo	Moderado
Norwood et al.^(17)^	Moderado	Baixo	Baixo	Baixo	Baixo	Moderado	Baixo	Moderado
Tsantefski et al.^(20)^	Moderado	Baixo	Baixo	Baixo	Baixo	Moderado	Baixo	Moderado
Bachi et al.^(21)^	Moderado	Baixo	Baixo	Baixo	Baixo	Moderado	Baixo	Moderado

**Legenda:** Baixo: baixo risco de viés; Moderado: Moderado risco de viés

**Quadro 5:** Análise da qualidade metodológica dos estudos randomizados - ROBINS 2

Estudo	Domínios						
	D1a	D1b	D2	D3	D4	D5	Geral
Hauge et al.^(29)^	+	+	+	+	+	+	+
Pendry et al.^(26)^	+	+	+	+	+	!	!

**Legenda:** D1a: processo de randomização; D1b: identificação e recrutamento dos participantes; D2: desvios na intervenção pretendida; D3: perda de dados; D4: medida do desfecho; Dd5: seleção dos resultados reportados. Cor verde e sinal de mais: baixo risco de viés; Cor amarela e exclamação: algumas preocupações quanto ao risco de viés

Nessa análise é possível observar que todos os estudos não randomizados apresentaram um risco de viés moderado, principalmente nos domínios de viés de confundimento e viés de medida dos desfechos. Na análise dos dois estudos randomizados incluídos na revisão, constatou-se que um apresentava baixo risco de viés e o outro apresentou algumas preocupações quanto ao risco de viés, sendo penalizado no domínio cinco, por uma falha no cegamento do estudo.

## DISCUSSÃO

Este estudo teve como objetivo investigar evidências da equitação e equoterapia em crianças e adolescentes neurotípicos, sendo possível observar que essas práticas apontaram melhoras em alguns aspectos cognitivos, de linguagem, comportamento, emocionais e sociais, embora a qualidade da evidência científica seja rebaixada uma vez que a maioria dos estudos são ensaios clínicos não randomizados (66%). Para responder questões relacionadas à intervenção “equitação”, o delineamento mais adequado, ou seja, aquele capaz de fornecer maior confiança nas informações encontradas, seria o ensaio clínico randomizado, e somente 33% dos estudos desta revisão o são.

Em relação aos desfechos encontrados na revisão, apenas o estudo não randomizado^(14)^ fez uma triagem de várias funções cognitivas. As habilidades de aritmética, atenção, percepção visual, funções executivas, linguagem oral e escrita, orientação, memória, fluência verbal e praxias foram investigadas, com melhoras significativas pré e pós intervenção nas cinco últimas habilidades citadas. Tais ganhos podem ser justificados com resultado de pesquisas sobre equitação e conectividade cerebral, os quais demonstram estreita relação da região talâmica bilateralmente com as funções superiores de atenção, linguagem, memória e função executiva^(34)^,e com possível ativação dessa área cerebral pós equitação^(6)^. Ademais, funções como “orientação” são complexas, podendo demandar integração entre atenção, memória e percepção.

A habilidade de memória refere-se à capacidade em adquirir, reter e usar conhecimentos e envolve diversos componentes corticais e subcorticais. A memória de trabalho é compreendida como um sistema de atenção limitada, no qual armazena informações somente enquanto uma tarefa está sendo realizada, por um período muito curto. As tarefas de atenção e memória de trabalho são interrelacionadas^(16)^. Estudos apontam que a equitação contribui para a ativação do lobo frontal e núcleo caudado à direita, estruturas responsáveis por essas habilidades^(6,35)^. O estudo não-randomizado^(17)^ também investigou sobre memória de trabalho e encontrou melhora significativa na reavaliação. Porém cada estudo^(14,17)^ utilizou um teste diferente para avaliar a memória de trabalho e o tempo de equitação variou, sendo um estudo com duração de cinco semanas^(17)^ e outro com duração de 12 meses^(14)^. Sabe-se que intervenções que se estendem por períodos mais longos tendem a proporcionar ganhos mais sustentáveis e significativos em habilidades cognitivas. Elas permitem reforçar e consolidar as habilidades adquiridas, facilitando a transferência desses ganhos para outras áreas da vida^(36)^.

Outro desfecho encontrado com ganhos significativos foi referente à linguagem oral e foi investigada essa habilidade somente em um estudo^(14)^. Estudos demonstram a relação entre o bom desempenho em linguagem oral e a competência em leitura^(37-41)^. Esse é um dado importante, visto que a população em situação de vulnerabilidade social apresenta baixo desempenho em leitura^(42)^. A linguagem oral sendo estimulada com a prática da equitação, pode ser um caminho para ganhos na aprendizagem e principalmente na leitura^(43).^

Em relação às funções executivas, em um estudo não randomizado^(14)^ foram investigadas as habilidades de resolução de problemas e fluência verbal, com melhora significativa apenas em fluência verbal, a qual trata-se de um processo executivo e envolve uma produção controlada de palavras a partir de uma regra semântica ou fonológica em um dado tempo^(16).^ Considerando a região cerebral responsável pela fluência verbal^(44)^, um estudo experimental comparou a conectividade cerebral funcional de crianças neurotípicas e com Transtorno do Déficit de Atenção antes e após quatro semanas da prática de equitação e apontou maior conectividade do giro frontal superior dos participantes neurotípicos^(6)^.

Em outro estudo não randomizado^(17)^, foi investigado o controle inibitório, outra habilidade dentro das funções executivas. A área cerebral responsável pela função executiva é o córtex pré-frontal bilateralmente e estudos apontam que essas habilidades aprimoram com o avanço da idade^(16,35)^.

Na habilidade de praxias as estruturas cerebrais ativadas são os lobos frontais e parietais, promovendo a discriminação e síntese visual, orientação espacial e outros pré-requisitos para desenvolvimento da escrita, desenho entre outros^(14,16)^. A equitação favorece as áreas do equilíbrio, coordenação motora grossa e fina e melhor conscientização corporal^(45-47)^.

Os estudos não randomizados realizados^(17,20)^, evidenciaram diminuição significativa estatisticamente da hiperatividade e o somente um estudo^(20)^ encontrou diminuição significativa estatisticamente de problemas emocionais e diminuição de comportamentos difíceis relatados pelos pais. Um estudo realizou a equoterapia^(20)^, com duração de 12 semanas e o outro estudo^(17)^ reforçou na metodologia que a intervenção seria sem terapeuta, somente a equitação e a interação homem-cavalo, com duração de 5 semanas. Estudos em equoterapia com pessoas com Transtorno do Déficit de Atenção e Hiperatividade, com duração de 12 semanas^(48-51)^, apontaram ganhos significativos nos aspectos comportamentais quanto à diminuição de problemas negativos e aumento de comportamentos positivos, redução da ansiedade, problemas sociais e hiperatividade, além da melhora da qualidade de vida. É discutida a importância da interação homem-cavalo, como a chave para a cura de problemas sociais e emocionais, pois o indivíduo pode se abrir e revelar seus sentimentos sem medo de julgamento^(50).^

Os dois estudos randomizados^(26,29)^ identificaram que após a intervenção com equitação, os indivíduos melhoraram significativamente a competência social e em outro^(26)^ os resultados apontaram diminuição dos comportamentos negativos e aumento dos comportamentos positivos estatisticamente significativos. Considera-se que a competência social está relacionada à capacidade de articular pensamentos, ações e sentimentos em função de demandas pessoais, da situação e da cultura que o indivíduo está inserido. Essa habilidade gera consequências positivas para o indivíduo e para sua relação com as pessoas, além de ser um fator importante para o ajustamento social e sucesso no ambiente escolar^(52)^. Nesse mesmo estudo é discutido que os problemas de comportamentos estão diretamente relacionados ao repertório reduzido de habilidades sociais e mau desempenho acadêmico das crianças e adolescentes. Estudos em equoterapia apontam melhora na ansiedade e do comportamento no geral em adolescentes em situação de risco^(53-55)^. Um estudo randomizado^(56)^ demonstrou diminuição dos níveis de cortisol em adolescentes após 11 semanas de equitação, sugerindo que a interação homem-cavalo diminui níveis de estresse, bem como a resposta ao estresse, sendo uma influência protetora contra o desenvolvimento de psicopatologias e problemas de saúde^(56).^

Um aspecto importante dos achados está relacionado ao número de estudos, pois observou-se que os não randomizados foram superiores aos randomizados, o que impacta diretamente na qualidade metodológica das pesquisas, que é importante no processo de gerar evidências científicas^(57)^. São necessários mais ensaios clínicos nessa área, tanto da equitação quanto na equoterapia, para a garantia da efetividade dessa prática no desenvolvimento humano. A diversidade metodológica dos estudos da revisão também dificultou a generalização dos achados, pois não foi possível compará-los.

Porém a evidência científica não se dá somente pela qualidade metodológica e também pela experiência clínica profissional e as preferências dos pacientes^(58)^. Realizar estudos que visam a Prática Baseada em Evidências (PBE) é de grande importância na clínica e na ciência, pois norteia o cuidado em saúde^(58)^ e consequentemente direcionam e transformam a prática clínica. Apesar dos artigos encontrados nessa revisão terem questões metodológicas a serem melhoradas, acredita-se que as pesquisas foram realizadas pelo fato da *expertise* clínica constatar os benefícios sociais, comportamentais, emocionais e cognitivos que a equitação proporciona para os indivíduos que realizam tal prática.

As revisões sistemáticas e as metanálises estão no topo da prática baseada em evidências e esta revisão de escopo teve como limitação importante o número insuficiente de ensaios clínicos randomizados com os mesmos desfechos investigados, para a realização dessa análise. Nessa revisão, cinco estudos apresentaram risco moderado de viés e um estudo randomizado apresentou baixo risco de viés. Sendo assim, a maioria não apresentou alta qualidade que pudesse comprovar o efeito observado. O rigor metodológico contribui para redução de vieses e resultados mais fidedignos. Nesse sentido, novos estudos podem modificar os desfechos observados.

A equitação é um esporte mais elitizado e de certa forma pouco acessível à população no geral. Para a realização de pesquisas nessa área, são necessárias pesquisas com financiamento por órgãos de fomento para que sejam recrutados participantes em número suficiente para um bom ensaio clínico randomizado. O grande entrave pode ser devido à carência de parcerias entre hípicas e universidades para o desenvolvimento de estudos com adequada qualidade metodológica. A partir da obtenção de evidências para equitação abre-se um novo horizonte com perspectivas também para a prática de equoterapia. Estudos de revisão sistemática com metanálise sobre a equoterapia com crianças com paralisia cerebral^(58)^ e com indivíduos com transtorno do espectro autista^(59)^ encontraram evidências científicas positivas dessa modalidade terapêutica, com esse público.

Em relação às limitações do presente estudo é possível destacar aspectos metodológicos das pesquisas encontradas, tais como ausência de grupo controle e da distribuição aleatória dos participantes, diferença na duração da intervenção, comprometendo a qualidade das evidências científicas encontradas.

## CONCLUSÃO

Embora os artigos não randomizados analisados tenham apresentado benefícios na realização da equitação nos desfechos referentes às funções cognitivas, de linguagem, aspectos comportamentais e emocionais, o fato das intervenções e mensurações dos desfechos não serem homogêneas, representa uma limitação para as conclusões do presente estudo. Importante ressaltar que os estudos randomizados dessa revisão, encontraram benefícios em relação à competência social após prática da equitação.
